# High Bone Sialoprotein (BSP) Expression Correlates with Increased Tumor Grade and Predicts a Poorer Prognosis of High-Grade Glioma Patients

**DOI:** 10.1371/journal.pone.0048415

**Published:** 2012-10-31

**Authors:** Tao Xu, Rong Qin, Jinxu Zhou, Yong Yan, Yicheng Lu, Xiaoping Zhang, Da Fu, Zhongwei Lv, Weiqing Li, Chunyan Xia, Guohan Hu, Xuehua Ding, Juxiang Chen

**Affiliations:** 1 Department of Neurosurgery, Shanghai Institute of Neurosurgery, Changzheng Hospital, Second Military Medical University, Shanghai, China; 2 Department of Pathology, Changzheng Hospital, Second Military Medical University, Shanghai, China; 3 Department of Nuclear Medicine, Shanghai 10th People’s Hospital, Tongji University School of Medicine, Shanghai, China; 4 The Key Laboratory of Stem Cell Biology, Institute of Health Sciences, Shanghai Institutes for Biological Sciences, Chinese Academy of Sciences/Shanghai JiaoTong University School of Medicine, Shanghai, China; The Ohio State University Medical Center, United States of America

## Abstract

**Objectives:**

To investigate the expression and prognostic value of bone sialoprotein (BSP) in glioma patients.

**Methods:**

We determined the expression of BSP using real-time RT-PCR and immunohistochemistry in tissue microarrays containing 15 normal brain and 270 glioma samples. Cumulative survival was calculated by the Kaplan-Meier method and analyzed by the log-rank test. Univariate and multivariate analyses were performed by the stepwise forward Cox regression model.

**Results:**

Both BSP mRNA and protein levels were significantly elevated in high-grade glioma tissues compared with those of normal brain and low-grade glioma tissues, and BSP expression positively correlated with tumor grade (*P*<0.001). Univariate and multivariate analysis showed high BSP expression was an independent prognostic factor for a shorter progression-free survival (PFS) and overall survival (OS) in both grade III and grade IV glioma patients [hazard ratio (HR) = 2.549 and 3.154 for grade III glioma, and HR = 1.637 and 1.574 for grade IV glioma, respectively]. Patients with low BSP expression had a significantly longer median OS and PFS than those with high BSP expression. Small extent of resection and lineage of astrocyte served as independent risk factors of both shorter PFS and OS in grade III glioma patients; GBM patients without *O^6^*-methylguanine (*O^6^*-meG) DNA methyltransferase (MGMT) methylation and Karnofsky performance score (KPS) less than 70 points were related to poor prognosis. Lack of radiotherapy related to shorter OS but not affect PFS in both grade III and grade IV glioma patients.

**Conclusion:**

High BSP expression occurs in a significant subset of high-grade glioma patients and predicts a poorer outcome. The study identifies a potentially useful molecular marker for the categorization and targeted therapy of gliomas.

## Introduction

Gliomas are the most common primary tumors in the central nervous system (CNS) and malignant gliomas, which account for 70% of gliomas, are the most frequent and lethal cancers originating in the CNS with a high recurrence and mortality rate [1]. The most biologically aggressive subtype of gliomas is glioblastoma multiforme (GBM) [World Health Organization (WHO) grade IV astrocytoma], a tumor associated with a rather dismal prognosis. Current standard treatment protocol includes maximal surgical resection followed by adjuvant chemotherapy with DNA alkylator, temozolomide, and radiotherapy. Despite the best available therapeutic regimen, the life expectancy of patients with GBM and anaplastic astrocytoma (WHO grade III) is still short, with a median survival of approximately only 14–16 months and 2–5 years, respectively [2].

Substantial efforts have been made in the identification of genetic alterations or molecular markers in GBMs that could help identify subgroups of GBM patients with differing prognoses or clinical response to specific therapies. One of the major determinants of clinical response to temozolomide is the methylation status of the promoter region of *O^6^*-methylguanine (*O^6^*-meG) DNA methyltransferase (MGMT). The hypermethylation of MGMT promoter is seen in approximately 40% to 60% of patients and correlates with a favorable response to temozolomide [3]. Recently, the gene encoding isocitrate dehydrogenase-1 (IDH1), which converts isocitrate to α-ketoglutarate by oxidative decarboxylation, has been found to be mutated in up to 75% of grade II and III diffuse gliomas, and IDH1 mutations have been found to be a major prognostic marker for overall and progression-free survival in grade II–IV gliomas [4].

**Table 1:** The demographic and clinicopathological characteristics of glioma patients.

Variables			Total(n = 270) n(%)	HGG(n = 162) n(%)
Age (years)				
		<65	228(84.4)	126(77.8)
		≥65	42(15.6)	36(22.2)
Gender				
		Male	177(62.1)	103(63.6)
		Female	193(32.6)	59(36.4)
Primary/Secondary				
		PrimaryGlioma	239(88.5)	137(84.6)
		SecondaryGlioma	31(11.5)	25(15.4)
Seizure				
		Yes	60(22.2)	25(36.4)
		No	210(77.8)	137(63.6)
IICP				
		Yes	103(38.1)	71(43.8)
		No	167(61.9)	91(56.2)
Cystic degeneration				
		Yes	60(22.2)	41(25.3)
		No	210(77.8)	121(74.7)
Necrosis on MRI				
		Yes	36(13.3)	31(19.1)
		No	234(86.7)	131(80.9)
MTD (cm)				
		<5	114(42.2)	65(40.1)
		≥5	156(57.8)	97(59.9)
Resection degree				
		>98%	208(77.0)	120(74.1)
		<98%	62(23.0)	42(25.9)
Tumor grade and pathological category				
	WHO Grade I		7(2.6)	
	Pilocyticastrocytoma		7	
	WHO Grade II		101(37.4)	
	Astrocytoma		67	
	Oligodendroglioma		27	
	Mixedoligo-astrocytoma		7	
	WHO Grade III		45(16.7)	45(27.8)
	Anaplasticastrocytoma		33	33
	Anaplasticoligodendroglioma		11	11
	Astroblastoma		1	1
	WHO Grade IV		117(43.3)	117(72.2)
	GBM		116	116
	Gliosarcoma		1	1
Radiotherapy				
	Yes		175(64.8)	116(71.6)
	No		95(35.2)	46(28.4)
Chemotherapy				
	Yes		174(64.4)	117(69.9)
	No		96(35.6)	45(30.1)
Lineage				
	Astrocytic		225(83.3)	151(93.2)
	Oligodendroglial		45(16.7)	11(6.8)
MGMT methylation				
	–		103(38.1)	79(48.5)
	+		167(61.9)	83(51.2)
KPS				
	<70		88(32.6)	73(45.1)
	≥70		182(67.4)	89(54.9)

Abbreviations: GBM, glioblastoma multiforme; HGG, high-grade glioma; IICP, increased intracranial pressure; MTD, mean tumor diameter.

High motility of cancer cells combined with increased expression of proteases that degrade the extracellular matrix is generally predictive of invasive capability. Bone sialoprotein (BSP), a specific regulator of matrix metalloproteinase-2 (MMP-2), is encoded by the *IBSP* gene and belongs to the small integrin-binding ligand *N*-linked glycoprotein (*SIBLING*) gene family [5], [6], [7]. BSP is highly expressed by numerous cancers such as prostate cancer, breast cancer, and pancreatic cancer and may contribute to their invasive potential [8]. An earlier study showed that BSP had statistically significant prognostic value in patients with clinically confined prostate adenocarcinomas [9]. Similar results were found in non-small cell lung cancer [10], breast cancer [11], [12], [13], human epithelial cancer [14], pancreatic cancer [15], and osteosarcoma [16]. However, there has been no study on the expression of BSP in glioma tissues and their importance as a prognostic predictor of glioma patients.

**Figure 1:**
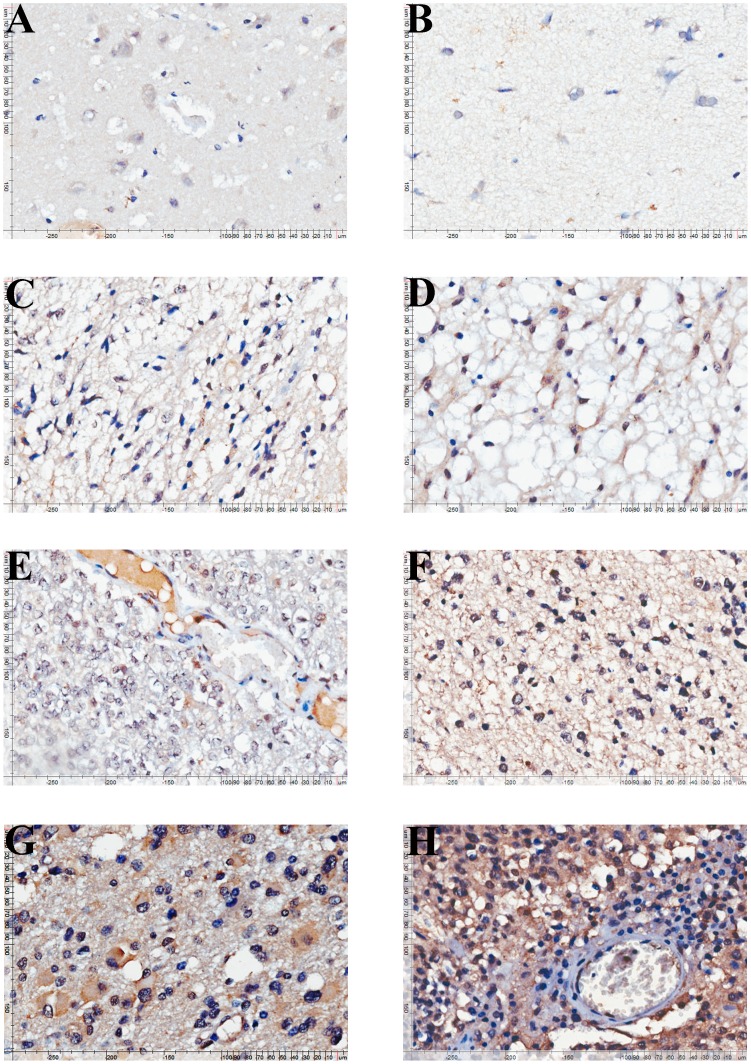
Survival and BSP expression pattern of glioma patients. The progression-free survival curve (A) and overall survival curve (B) in all 270 glioma patients (indicated in black) and 162 high-grade glioma patients (indicated in red). (C) Quantitative RT–PCR assays and (D) immunohistochemical staining showed that high-grade glioma (HGG) tissues expressed significantly higher levels of BSP mRNA transcripts and protein than low-grade glioma (LGG) and normal brain (NB) tissues. Data is expressed as Mean ± SE and the differences among groups were determined using Mann-Whitney U test (^#^ *P*>0.05, LGG vs. NB).

Our survey of some published microarray databases in Oncomine (www.oncomine.org) found that 2 datasets showed a higher expression of BSP in glioma tissues compared with the normal brain. Five other datasets showed that the expression of BSP was significantly higher in GBM than gliomas of other grades. In an attempt to further identify molecular markers in gliomas that help define subgroups of glioma patients with differing prognoses, we carried out preliminary Affymetrix U133 expression array analysis of 20 fresh snap-frozen glioma samples including 8 high-grade glioma (HGG) and 12 low-grade glioma (LGG) tissue samples and one normal brain sample. The study found significantly elevated mRNA transcript levels of BSP in glioma tissues, especially in HGG tissues (unpublished data). These results indicate that the expression of BSP may become dysregulated in human glioma, especially GBM, and could be of use as a molecular marker for determining the prognosis of glioma patients.

**Figure 2:**
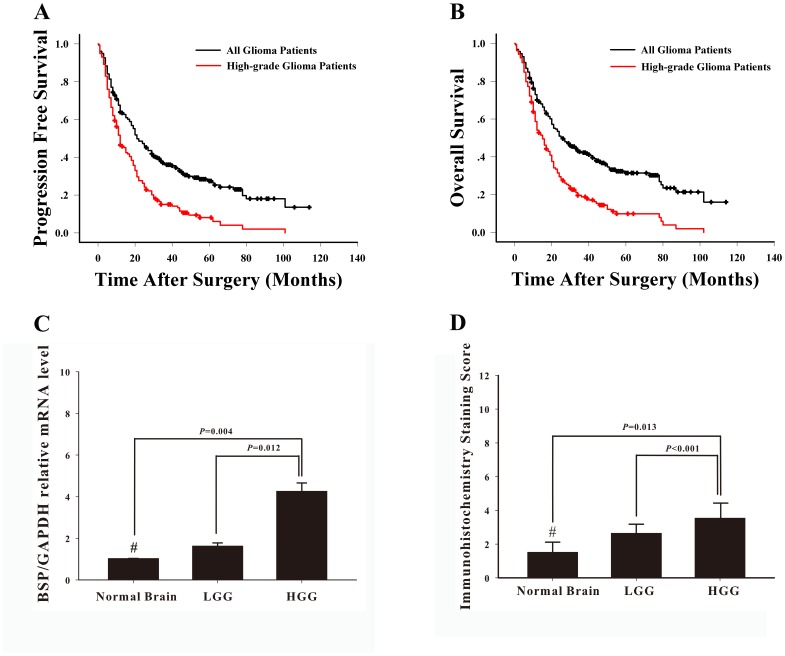
Immunohistochemical staining of BSP in glioma and normal brain tissues. Immunohistochemical staining of BSP in normal brain tissue (A, B, staining intensity score = 2), grade I pilocytic astrocytoma (C, staining intensity score = 3), grade II astrocytoma (D, staining intensity score = 3), grade II oligodendroglioma (E, staining intensity score = 3), grade III anaplastic oligodendroglioma (F, staining intensity score = 6) and grade III anaplastic astrocytoma (G, staining intensity score = 6) and grade IV (H, staining intensity score = 9) glioma tissue specimens.

In the current study, we sought to examine the expression of BSP through real-time reverse-transcription polymerase chain reaction (RT-PCR) and immunohistochemistry in tissue microarray so as to investigate the correlation between BSP expression and the clinicopathological characteristics of glioma patients. Our study has shown that BSP is overexpressed in certain glioma tissues and high BSP expression correlates with tumor grade and predicts a poorer survival of glioma patients.

## Patients and Methods

### Acquisition of Tissue Specimens

The study protocol and acquisition of tissue specimens were approved by the Specialty Committee on Ethics of Biomedical Research, Second Military Medical University (SMMU), Shanghai, China. Glioma tissue specimens were obtained from archived tissue samples of patients who underwent surgical treatment at the Department of Neurosurgery in Changzheng Hospital, SMMU, between January 1999 and December 2008. Normal brain tissue specimens were obtained from trauma patients for whom partial resection of normal brain was required as decompression treatment for severe head injuries. These patients or their legal surrogates provided written informed consent to the surgical procedures and gave permission to the use of resected tissue specimens. The selection criteria of this study were as follows: 1) the subject had a primary diagnosis of glioma and no history of other tumors; 2) the subject had complete clinical data, including age, gender, clinical manifestations, mean tumor diameter (MTD, defined as the geometric mean of the 3 diameters on MRI scan), extent of resection, histological type, pathological grade and adjuvant therapy; 3) the subject underwent evaluation by enhanced head MRI scans for tumor relapse or progression after surgery at least once every six months; 4) the tissue sample was of sufficient quality for experimental use.

**Table 2:** Univariate analysis of factors associated with survival and progression in Grade III and IV glioma patients.

Variable	Grade III						Grade IV					
	OS			PFS			OS			PFS		
	HR	95% CI	P	HR	95% CI	P	HR	95% CI	P	HR	95% CI	P
Age (≥65 vs. <65 y)	1.397	0.563–3.466	0.471	1.396	0.560–3.482	0.474	1.470	0.946–2.286	0.087	1.515	0.976–2.353	0.064
BSP (High vs. low)	2.451	1.143–5.258	0.021	2.274	1.063–4.861	0.034	1.672	1.111–2.515	0.014	1.715	1.142–2.575	0.009
Chemotherapy (Yes vs. no)	0.804	0.385–1.679	0.562	0.909	0.441–1.874	0.797	0.666	0.429–1.034	0.070	0.780	0.502–1.211	0.269
IICP (Yes vs. no)	0.870	0.425–1.782	0.703	0.908	0.454–1.816	0.785	0.870	0.425–1.782	0.703	0.828	0.565–1.214	0.334
MTD (≥5 vs <5 cm)	1.525	0.694–3.349	0.294	1.677	0.794–3.542	0.175	0.930	0.634–1.364	0.711	1.187	0.808–1.744	0.383
KPS (≥70 vs. <70)	0.700	0.300–1.636	0.410	0.666	0.287–1.545	0.343	0.734	0.465–1.157	0.182	0.604	0.382–0.957	0.032
Necrosis on MRI (Yes vs. no)	1.137	0.457–2.833	0.782	1.159	0.472–2.847	0.748	0.883	0.536–1.457	0.627	1.194	0.739–1.930	0.468
Primary (vs. secondary)	0.599	0.249–1.443	0.253	0.548	0.231–1.303	0.174	0.955	0.543–1.680	0.874	1.249	0.719–2.169	0.431
Radiotherapy (Yes vs. no)	0.483	0.233–1.002	0.051	0.607	0.300–1.229	0.165	0.658	0.430–1.005	0.053	0.692	0.453–1.057	0.089
Resection Degree (>98% vs.<98%)	1.943	1.031–3.659	0.040	1.939	0.887–4.237	0.097	1.154	0.754–1.766	0.508	1.095	0.719–1.668	0.673
Seizure (Yes vs. no)	1.201	0.481–3.000	0.695	1.277	0.544–2.997	0.574	0.864	0.482–1.547	0.622	1.098	0.623–1.934	0.746
MGMT(Methylation − vs. +)	2.288	1.076–4.867	0.032	2.234	1.077–4.634	0.031	1.537	1.020–2.314	0.040	1.418	0.947–2.123	0.090
Lineage (Oligodendroglial vs. astrocytic)	0.405	0.140–1.171	0.095	0.467	0.178–1.223	0.121	NA	NA	NA	NA	NA	NA

NOTE. Univariate analysis, Cox proportional hazards regression model. Abbreviations: IICP, increased intracranial pressure; MTD, mean tumor diameter; HR, Hazard ratio; OS, overall survival; PFS, progression-free survival; NA, not applicable.

**Figure 3:**
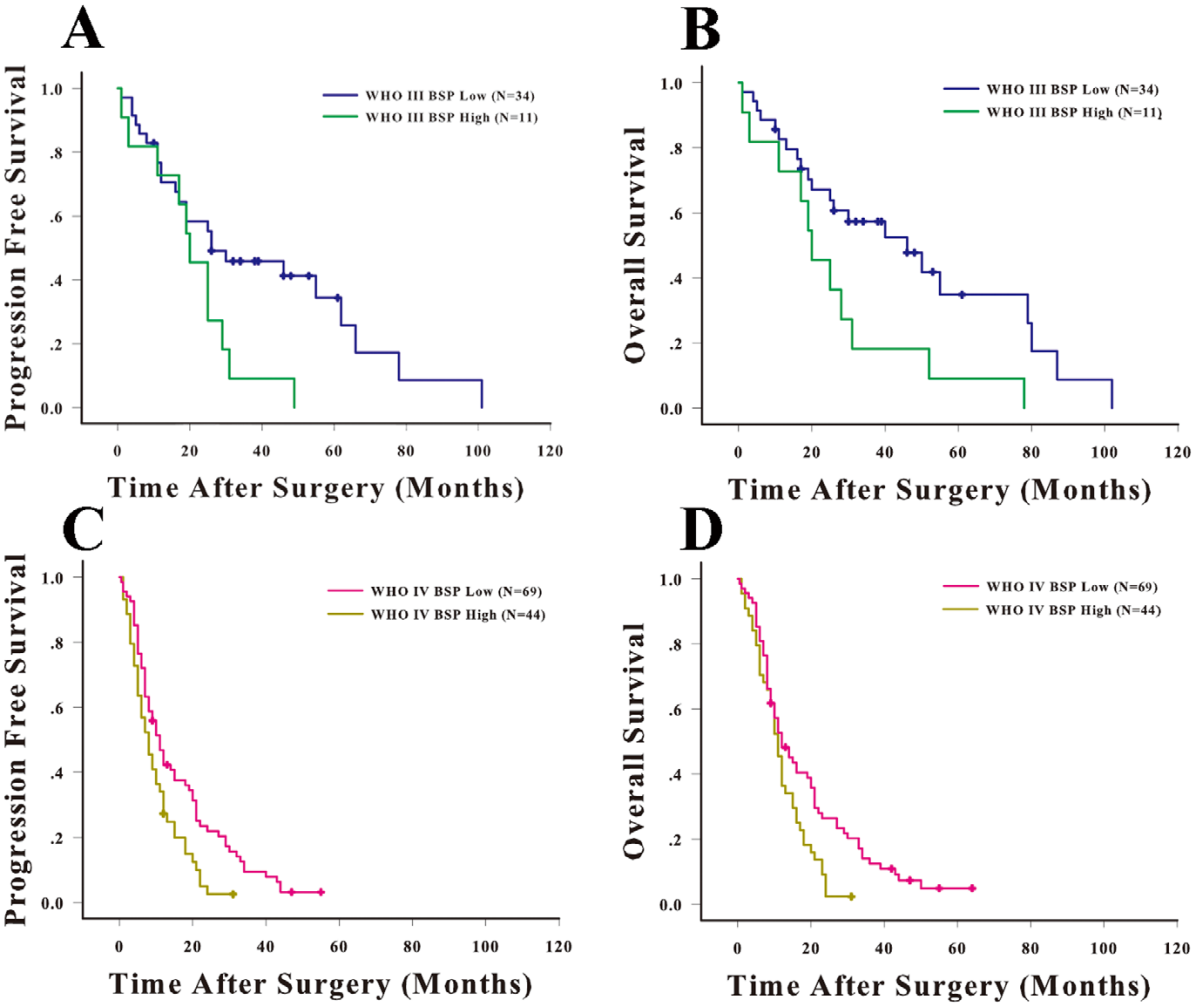
High BSP expression is associated shorter overall survival and progression-free survival of WHO grade III and IV glioma patients. The progression-free survival (A) and Kaplan-Meier survival curve (B) for WHO grade III glioma patients stratified by BSP expression. The progression-free survival (C) and Kaplan-Meier survival curve (D) for WHO grade IV glioma patients stratified by BSP expression.

**Figure 4:**
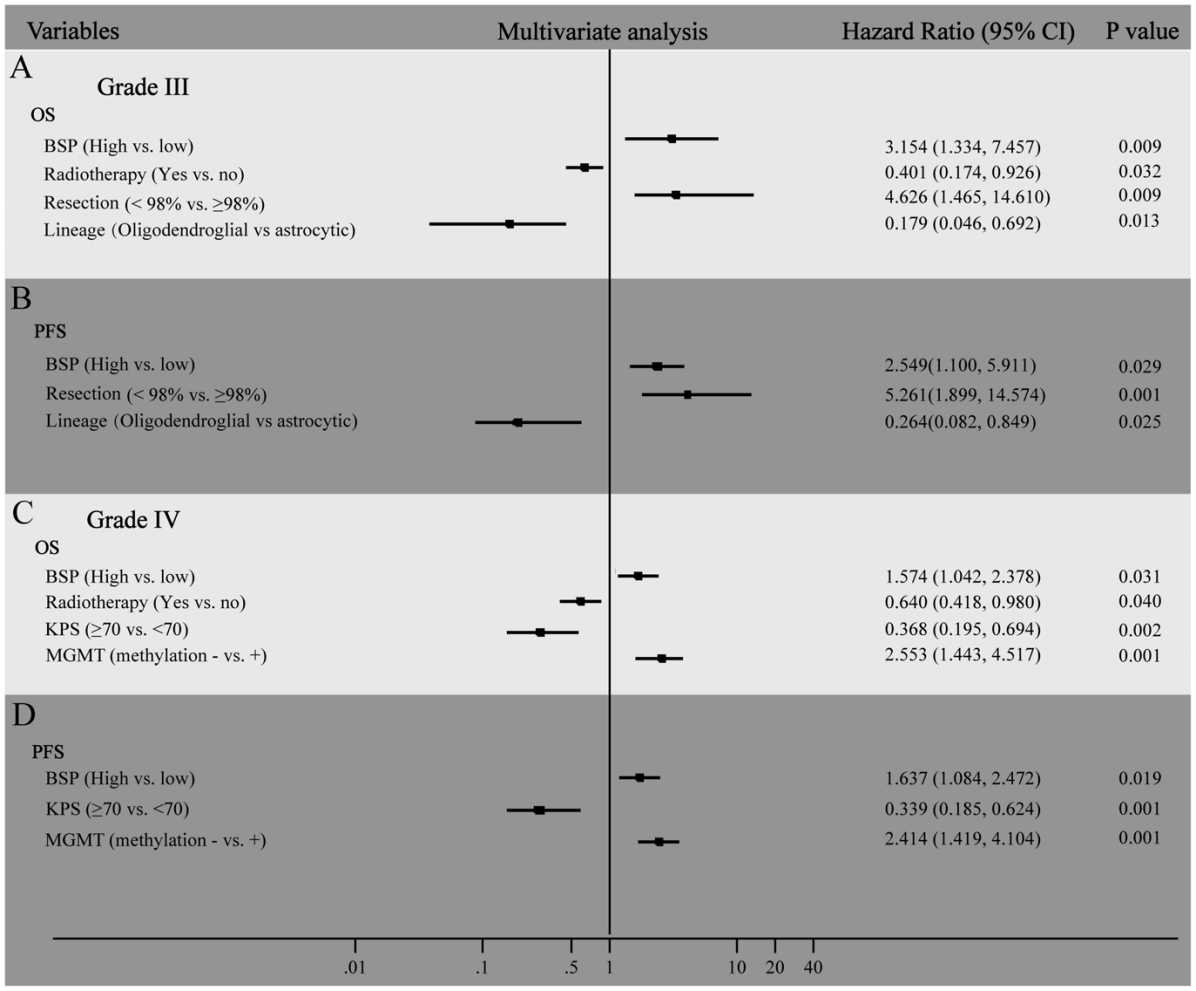
Multivariate analysis of factors associated with survival and progression of WHO grade III and IV glioma patients. Hazard ratio and 95% CIs for factors that related to OS (A) and PFS (B) in WHO grade III glioma patients, OS (C) and PFS (D) in WHO grade IV glioma patients.

These patient samples were divided into two groups, with no crossovers with each other. All the pathological diagnoses were reviewed by two experienced pathologists independently, according to the 2007 WHO classification of tumors of the central nervous system.

In the first group, the fresh frozen tissues from 25 LGG patients, 30 HGG patients and 7 normal brain samples were obtained during the operations for real-time RT-PCR.

In the second group, paraffin embedded tissue samples from 300 patients with different grades of glioma undergoing surgical resections were obtained, 16 normal brain tissues were obtained from surgical resections of trauma patients, for whom a partial resection of normal brain tissue was required as decompression treatment for their severe head injuries to reduce increased intracranial pressure under the permission of each of the patient’s family. The tissue microarray (TMA) was constructed based on these samples.

### Real-time RT-PCR

62 fresh snap-frozen tissues from 25 LGG, 30 HGG and 7 normal brain samples were employed for isolation of total RNA using the Trizol reagent (Invitrogen, Carlsbad, CA) according to the manufacturer’s instructions. Total RNA (2 µg) was then reverse transcribed using the *M-MLV* Reverse Transcriptase Kit (Promega, Madison, WI). The resultant cDNA (20 ng) was mixed with SYBR GreenMasterMix (BioRad, Hercules, CA) and amplified in the CFX96 real-time detection system (Bio-Rad). Each reaction was run in triplicates. The expression of *IBSP* was normalized against *GAPDH* by the comparative threshold cycle (ct) method using the following formula: fold difference in expression = 2^–(Δct of target gene–Δct of reference)^. The following primers were used for RT-PCR: *IBSP*, 5′-AAAGTGAGAACGGGGAACCTR-3′ (sense) and 5′-GATGCAAAGCCAGAATGGAT-3′ (antisense) and *GAPDH*, 5′-GAAGGTGAAGGTCGGAGTC-3′ (sense) and 5′-GAAGATGGTGATGGGATTTC-3′ (antisense).

### Tissue Microarray Construction and Immunohistochemistry

Paraffin-embedded tissues were acquired from 275 patients with different grades of glioma and from 16 trauma patients for whom partial resection of normal brain tissue was required. Tissue microarrays of these sample specimens were constructed as described previously in Shanghai Biochip Co., Shanghai, China [17]. Rabbit polyclonal anti-human BSP antibodies were purchased from Abcam Co., Ltd (Cambridge, MA), the immunohistochemical staining were performed according to the manufacturer’s instructions. The sections not incubated with the primary antibody were used as negative controls and sections from a patient of non-small lung cancer was used as positive controls.

The immunohistochemical results were evaluated by two independent pathologists with no knowledge of the demographic and clinicopathological data of the patients. For glioma cells, staining in the cytoplasms of neoplastic cells were regarded as positive, while for normal brain, only staining in the cytoplasms of astrocytes and oligodendrocytes were regarded as positive. The intensity of positive staining was scored using a scale from 0 to 3 (0, no immunostaining; 1, light brown color; 2, medium brown color; and 3, dark brown color). The percentage of positive staining cells was also scored (0, no staining; 1, positive staining in <25% of the tumor cells; 2, positive staining in 25–75% of the tumor cells; and 3, positive staining in >75% of the tumor cells.). The percentage of cells showing positive staining with the antibodies was calculated in 5 high-powered fields. The two scores were then multiplied, and the results were regarded as the expression score of the sample. All discrepancies in scoring were reviewed, and a consensus was reached. Samples were scored totally as follows: strong (+++, total score >6), moderate (++, total score = 4∼6), weak (+, total score = 1∼3), and null (-, total score = 0). BSP was recorded as high expression (++ and +++), and low or negative expression (+ and -) according to the rate of labeled tumor cells and cytoplasm staining intensity.

### Statistical Analysis

Overall survival (OS) and the progression-free survival (PFS) were recorded for each patient. PFS was defined as the time from initial surgical diagnosis to tumor progression in MRI or death from glioma. OS was defined as the time from the initial surgical diagnosis to death. Mann-Whitney U test was used to compare the mRNA expression and immunostaining scores of BSP in HGG, LGG and normal brain tissues. Chi square test was used to compare BSP expression with other clinicopathological factors. Cumulative survival was calculated by the Kaplan-Meier method and analyzed by log-rank test. Univariate and multivariate analyses were performed by stepwise backward Cox regression model (*P*<0.2 was considered as the inclusion criterion for factors that could be added into multivariate analysis). All statistical analyses were performed with the SPSS 16.0 software (SPSS Inc, Chicago, IL), *P*<0.05 was considered as statistically significantly different.

## Results

### Demographic and Clinicopathological Characteristics of Study Subjects

We performed tissue microarray of 275 glioma tissue samples and 16 normal brain tissue samples. Among the glioma patients, 270 glioma (including 108 LGG and 162 HGG) and 15 normal brain tissue samples met our criteria for further evaluation (the other 6 tissue dots were lost from the TMA slide). The demographic and clinicopathological characteristics of these patients are listed in Table 1. Sixty percent of the patients (162/270) had HGG and approximately 3/4 of them (72.2%, 117/162) had WHO grade IV glioma.

258 patients (95.6%) were followed up from 0.3 to 114 months. The median PFS was 21 months (95% CI, 16.72–25.28 months) and, for HGG patients, 12 months (95% CI, 9.18–14.82 months). The PFS rates for HGG patients were 51% at 1 year, 28% at 2 years, and 8% at 5 years, respectively (Figure 1A). At the final follow-up, 28% (28/100) LGG patients and 87.3% (138/158) HGG patients died. The median OS was 26 months (95% CI, 18.89–33.11 months). The OS rates for HGG patients were 58% at 1 year, 32% at 2 years, and 10% at 5 years, respectively, with a median OS of 15 months (range, 11.74–18.26 months). The Kaplan-Meier survival curve is shown in Figure 1B.

### BSP Expression Correlates with Glioma Grade

Our preliminary Affymetrix U133 expression arrays indicated significantly elevated mRNA transcript levels of BSP in glioma tissues, especially HGG tissues (Unpublished data). This result was verified using RT-PCR assays in this study. We found that HGG tissues expressed significantly higher mRNA transcript levels of BSP than LGG or normal brain tissues (*P* = 0.012 and *P* = 0.004, respectively) (Figure 1C).

We then examined the expression of BSP in glioma and normal brain tissues by immunohistochemistry using tissue microarray (Figure 2). Immunohistochemical staining showed markedly elevated expression of BSP in HGG tissues compared with that of LGG or normal brain tissues (P<0.001 and P = 0.013, respectively) (Figure 1D). By contrast, no significant difference was noted in BSP mRNA transcript levels or protein levels between LGG and normal brain tissues (P>0.05). We found that about three quarters of glioma tissue specimens (73.33%, 198/270) expressed low levels of BSP while approximately one fourth (26.67%, 72/270) exhibited high BSP expression (Table S1). Furthermore, 35.2% (57/162) of HGG tissue specimens showed high BSP expression while only 13.9% (15/108) of LGG tissue specimens exhibited high BSP expression (*P*<0.001). In addition, the high BSP expression rate increased with tumor grade: approximately 40% (39.32%, 46/117) grade IV glioma tissue specimens showed high BSP expression while only 13.9% (15/108) grade I–II glioma tissue specimens exhibited high BSP expression (Table S1), a finding further confirmed by our correlation analysis which revealed that high BSP expression correlated with higher tumor grade (*P*<0.001).

### Correlation of BSP with Other Clinical Characteristics

The characteristics of glioma patients with high BSP expression are shown in Table S1. To assess whether BSP expression correlated with clinicopathological characteristics other than tumor grade, we did a correlation analysis and found that high BSP expression correlated with older age (*P* = 0.007), absence of seizure (*P* = 0.001), and astrocytic tumor lineage (*P* = 0.003), but did not correlate with any other clinicopathological parameters (Table S1). In HGG patients, high BSP expression also correlated with a larger MTD (*P* = 0.021). Since these factors are known to be associated with increased invasiveness of human glioma [18], [19], [20], [21], the results indicated that BSP high-expression might be associated with poorer prognosis of human glioma patients independent of tumor grades.

### High BSP Expression Correlates with Poorer Survival of Grade III and IV Glioma Patients

The prognostic effects of high BSP expression in patients with different grades of glioma were evaluated seperately. OS and PFS were stratified by BSP expression using the log-rank and Cox regression test.

In grade I and II glioma patients, high BSP expression did not correlate with shorter PFS and OS (P>0.05). However more than 70% LGG patients were alive and progression free at the last follow-up, so the prognostic value of BSP cannot be accurately predicted in the current study, thus require future studies with a follow-up period of sufficient duration that allows the analysis of such endpoints as recurrence or death.

In grade III glioma patients, we found that the median PFS of those patients with low BSP expression was 30 months, which was significantly longer that of HGG patients with high BSP expression (20 months) (*P* = 0.027) (Figure 3A). The median OS of patients with low BSP expression was 46 months, which was significantly longer that of HGG patients with high BSP expression (20 months) (*P* = 0.017) (Figure 3B). The univariate analysis showed high BSP expression was a potential risk factor of survival and progression of grade III glioma patients (HR = 2.274, Table 2). In the multivariate Cox regression, we found high-expression of BSP, small extent of resection and lineage of astrocyte as independent risk factors of poor prognosis in grade III glioma patients, while lack of radiotherapy only related to shorter OS but not affect PFS (Figure 4A and B). The hazard ratio for progression among patients with high BSP expression, as compared to those with low BSP expression, was 2.549 (95% CI, 1.100 to 5.911; *P* = 0.029). The hazard ratio for death among patients with high BSP expression, as compared to those with low BSP expression, was 3.154 (95% CI, 1.334 to 7.457; *P* = 0.009).

In grade IV glioma patients, we found that the median PFS of those patients with low BSP expression was 11 months, which was longer that of GBM patients with high BSP expression (8 months) (*P* = 0.006) (Figure 3C). The median OS of patients with low BSP expression was 12 months, which was significantly longer that of GBM patients with high BSP expression (11 months) (*P* = 0.01) (Figure 3D). The univariate analysis showed high BSP expression was a potential risk factor of survival and progression of grade IV glioma patients (HR = 1.672 and 1.715, respectively). In the multivariate Cox regression, we found high-expression of BSP, no MGMT methylation, low KPS were independent risk factors of poor prognosis (both OS and PFS) in grade IV glioma patients, while lack of radiotherapy only related to shorter OS but not affect PFS (Figure 4C and D). The hazard ratio for progression among patients with high BSP expression, as compared to those with low BSP expression, was 1.637 (95% CI, 1.084 to 2.473; *P* = 0.019). The hazard ratio for death among patients with high BSP expression, as compared to those with low BSP expression, was 1.574 (95% CI, 1.042 to 2.378; *P* = 0.031). The cumulative events of two groups (high BSP and low BSP) at certain time periods were listed in Table S2.

We further stratified HGG patients by both BSP expression and tumor grade. Patients with WHO grade IV and high BSP level had the significantly shortest median PFS (8 months) and OS (11 months), while those with WHO grade III and low BSP level had the significantly longest median PFS (30 months) and OS (46 months). There was no difference in PFS and OS between patients with WHO grade IV, low BSP level and patients with WHO grade III, high BSP level (Table S3). The results established an independent prognostic role for BSP in HGG patients.

## Discussion

In the present study, we examined the expression of BSP by RT-PCR and immunohistochemistry using tissue microarray and found that BSP was highly expressed in a significant subset of HGG tissues. The finding confirms the results of our preliminary Affymetrix U133 expression array analysis, which revealed significantly elevated mRNA transcript levels of BSP in HGG tissues. Our finding is also consistent with the data of some published microarray databases (www.oncomine.org) showing significantly higher BSP expression in GBM than in normal brain (6.5–9.4 folds change) or other types of glioma (2.7–7.2 folds change). Our Kaplan-Meier study and correlation analysis further demonstrated that high BSP expression was associated with a significant shorter PFS and OS. No previous publication is available on the expression of BSP in glioma tissues and its significance for glioma patients. Our study here provides the first evidence that BSP expression is dysregulated in a significant subset of malignant glioma patients and correlates with survival of glioma patients.

As HGG patients have significantly reduced OS compared with LGG patients [1] and a higher proportion of our HGG patients expressed high levels of BSP, it remains a distinct possibility that the significantly shorter PFS and OS in our patients expressing high levels of BSP may be simply due to the overall higher tumor grade in these patients. However, when we stratified HGG patients and primary GBM patients by BSP expression, separately, we found that the PFS and OS of HGG patients and primary GBM patients expressing high levels of BSP were considerably reduced compared with those expressing low levels of BSP, suggesting that BSP is an independent predictor of the therapeutic outcome of HGG patients. LGG patients have a better prognosis than HGG patients and have a median survival of almost 84 months [22]. In the current study, most LGG patients were alive and progression free at the last follow-up, so we cannot accurately predict the prognostic value of BSP for OS in LGG patients right now, which may require future studies with a follow-up period of sufficient duration that allows the analysis of such endpoints as recurrence or death. Nevertheless, LGG patients expressing high levels of BSP still showed a relatively higher progression rate (36.0% versus 57.1%) and a shorter PFS (27 months versus 78 months), indicating that LGG patients expressing high levels of BSP likely fare worse in outcome than their counterparts expressing low levels of BSP.

The molecular mechanisms whereby BSP promotes tumor progression have been addressed by several investigators. BSP can bind to and modulate the activity of MMP2, thus activating the MMP pathway [23]. It also accelerates the proliferation, invasion, and metastasis of cancer cells [24]. BSP may also act as a pro-angiogenic factor to promote angiogenesis through binding to integrin αvβ3 [25]. However, the role of BSP in gliomagenesis and progression of glioma remains unknown. The serum content of BSP in glioma patients and its correlation with the prognosis and therapeutic response of glioma patients also remain to be investigated. Serum BSP has been shown to predict bone metastasis of primary breast cancer [26] and survival and bone metastasis of prostate cancer patients [27]. It is of clinical importance to investigate whether serum BSP can serve as a convenient molecular marker to define the subset of glioma patients at risk for progression or poor survival. Patients with WHO grade II or III glioma could progress to WHO grade IV GBM (secondary GBM) upon recurrence. There is also the possibility that a fraction of GBMs designated as primary tumors may follow a sequence of genetic events similar to that of secondary GBM but do not come to clinical attention until malignant progression to a GBM has occurred. So far, there are no good methods to distinguish primary and secondary GBMs as the histopathological findings of primary and secondary GBMs are indistinguishable. Though we have established in the current study that glioma tissues expressing high levels of BSP are at risk for tumor recurrence independent of tumor grade, we do not have sufficient data on the expression of BSP in primary and secondary GBMs, which remain a critical issue to be addressed in future studies. What’s more, since BSP links closely with CD44, which is an important molecular biomarker of Mesenchymal subtype of glioblastoma(2), we hypothesis that BSP might serve as a diagnostic biomarker and a potential therapeutic target for this special subtype of glioblastoma. Further investigations are warranted in this field.

In conclusion, we demonstrate that high BSP expression is associated with increased tumor grade and shorter PFS and OS after surgery and BSP could help define the subset of glioma patients at higher risk for progression and poorer prognosis, thus serving as an independent prognostic factor for glioma patients. In addition, understanding the molecular pathways involved by BSP could result in the development of novel, tailored pharmacological therapies for human glioma patients.

## Supporting Information

Table S1 **Correlation Between the Expression Level of BSP and Clinicopathalogic Characteristics in All 270 Patients and 162 HGG Patients in TMA.**
(DOC)

Table S2 **Cumulative events for PFS and OS in two groups (high BSP and low BSP) at certain time periods.**
(DOC)

Table S3 **Comparison of progression free survival time and overall survival time among different groups of patients when stratified by BSP expression and tumor grade.**
(DOC)
