# Supplementary material for: High Bone Sialoprotein (BSP) Expression Correlates with Increased Tumor Grade and Predicts a Poorer Prognosis of High-Grade Glioma Patients
Source: PLoS One. 2012 Oct 31;7(10):e48415. doi: 10.1371/journal.pone.0048415 (PMC3485236; doi:10.1371/journal.pone.0048415)
Supplement: Table S1 — Correlation Between the Expression Level of BSP and Clinicopathalogic Characteristics in All 270 Patients and 162 HGG Patients in TMA. (DOC) [file pone.0048415.s001.doc]

| **Table S1**. **Correlation Between the Expression Level of BSP and Clinicopathalogic Characteristics in All 270 Patients and 162 HGG Patients in TMA** | | | | | | | |
| --- | --- | --- | --- | --- | --- | --- | --- |
| Variable | Total | | |  | HGG | | |
|  | Low | High (%) | P |  | Low | High (%) | P |
| Age |  |  |  |  |  |  |  |
| ＜65 y | 174 | 54(23.7) | 0.007 |  | 86 | 40(31.7) | 0.086 |
| ≥65 y | 24 | 18(42.9) |  |  | 19 | 17(47.2) |  |
| Gender |  |  |  |  |  |  |  |
| Male | 131 | 46(26.0) | 0.728 |  | 66 | 37(35.9) | 0.795 |
| Female | 67 | 26(28.0) |  |  | 39 | 20(33.9) |  |
| Primary/Secondary |  |  |  |  |  |  |  |
| Primary Glioma | 175 | 64(26.8) | 0.908 |  | 87 | 50(36.5) | 0.413 |
| Secondary Glioma | 23 | 8(25.8) |  |  | 18 | 7(28.0) |  |
| Seizure |  |  |  |  |  |  |  |
| Yes | 54 | 6(10.0) | 0.001 |  | 21 | 4(16.0) | 0.029 |
| No | 144 | 66(31.4) |  |  | 84 | 53(38.7) |  |
| IICP |  |  |  |  |  |  |  |
| Yes | 72 | 31(30.1) | 0.317 |  | 45 | 26(36.6) | 0.736 |
| No | 126 | 41(24.6) |  |  | 60 | 31(34.1) |  |
| Cystic degeneration |  |  |  |  |  |  |  |
| Yes | 42 | 18(30.0) | 0.508 |  | 26 | 15(36.6) | 0.828 |
| No | 156 | 54(25.7) |  |  | 79 | 42(34.7) |  |
| Necrosis on MRI |  |  |  |  |  |  |  |
| Yes | 22 | 14(38.9) | 0.075 |  | 19 | 12(38.7) | 0.648 |
| No | 176 | 58(24.8) |  |  | 86 | 45(34.4) |  |
| MTD |  |  |  |  |  |  |  |
| ＜5 cm | 88 | 26(22.8) | 0.938 |  | 49 | 16(24.6) | 0.021 |
| ≥5 cm | 110 | 46(29.5) |  |  | 56 | 41(42.3) |  |
| Grade |  |  | ＜0.001 |  |  |  |  |
| WHO I | 5 | 2(28.6) |  |  |  |  |  |
| WHO II  WHO III  WHO IV | 88  34  71 | 13(12.9)  11(24.4)  46(39.3) |  |  | 34  71 | 11(24.4)  46(39.3) | 0.076 |
| Lineage |  |  |  |  |  |  |  |
| Astrocytic | 157 | 68(30.2) | 0.003 |  | 94 | 57(37.7) | 0.011 |
| Oligodendroglial | 41 | 4(8.9) |  |  | 11 | 0(0.0) |  |
| **Abbreviations: TMA, tissue microarray; IICP, increased intracranial pressure; MTD, mean tumor diameter;** | | | | | | | |
