# Supplementary material for: High Bone Sialoprotein (BSP) Expression Correlates with Increased Tumor Grade and Predicts a Poorer Prognosis of High-Grade Glioma Patients
Source: PLoS One. 2012 Oct 31;7(10):e48415. doi: 10.1371/journal.pone.0048415 (PMC3485236; doi:10.1371/journal.pone.0048415)
Supplement: Table S2 — Cumulative events for PFS and OS in two groups (high BSP and low BSP) at certain time periods. (DOC) [file pone.0048415.s002.doc]

**Table S2a. Cumulative events for PFS in two groups (high BSP and low BSP) at certain time periods**

| **Months** | **0** | **12** | **24** | **36** | **48** | **48+** |
| --- | --- | --- | --- | --- | --- | --- |
| **WHO III/BSP low patients** | **34** | **24** | **19** | **12** | **8** | **0** |
| **Cumulative events** | **0** | **8** | **13** | **17** | **18** | **23** |
| **WHOIII/BSP high patients** | **11** | **8** | **5** | **1** | **1** | **0** |
| **Cumulative events** | **0** | **3** | **6** | **10** | **10** | **11** |
| **WHO IV/BSP low patients** | **69** | **32** | **15** | **6** | **1** | **0** |
| **Cumulative events** | **0** | **36** | **52** | **61** | **65** | **65** |
| **WHO IV/BSP high patients** | **44** | **15** | **2** | **0** | **-** | **-** |
| **Cumulative events** | **0** | **29** | **41** | **42** | **-** | **-** |

**Table S2b. Cumulative events for OS in two groups (high BSP and low BSP) at certain time periods.**

| **Months** | **0** | **12** | **24** | **36** | **48** | **48+** |
| --- | --- | --- | --- | --- | --- | --- |
| **WHO III/BSP low patients** | **34** | **26** | **21** | **14** | **9** | **0** |
| **Cumulative events** | **0** | **6** | **10** | **13** | **15** | **21** |
| **WHOIII/BSP high patients** | **11** | **8** | **5** | **2** | **2** | **0** |
| **Cumulative events** | **0** | **3** | **6** | **9** | **9** | **11** |
| **WHO IV/BSP low patients** | **69** | **36** | **17** | **9** | **3** | **0** |
| **Cumulative events** | **0** | **32** | **50** | **58** | **62** | **63** |
| **WHO IV/BSP high patients** | **44** | **20** | **4** | **0** | **-** | **-** |
| **Cumulative events** | **0** | **24** | **40** | **43** | **-** | **-** |
