# Supplementary material for: High Bone Sialoprotein (BSP) Expression Correlates with Increased Tumor Grade and Predicts a Poorer Prognosis of High-Grade Glioma Patients
Source: PLoS One. 2012 Oct 31;7(10):e48415. doi: 10.1371/journal.pone.0048415 (PMC3485236; doi:10.1371/journal.pone.0048415)
Supplement: Table S3 — Comparison of progression free survival time and overall survival time among different groups of patients when stratified by BSP expression and tumor grade. (DOC) [file pone.0048415.s003.doc]

**Table S3a Comparison of progression free survival time among different groups of patients when stratified by BSP expression and tumor grade**

| **Group**  **(Median PFS)** | **WHO III BSP Low**  **(30 months)** | **WHO III BSP High**  **(20 months)** | **WHO IV BSP Low**  **(11 months)** | **WHO IV BSP High**  **(8 months)** |
| --- | --- | --- | --- | --- |
| **WHO III BSP Low** |  |  |  |  |
| **WHO III BSP High** | **0.027** |  |  |  |
| **WHO IV BSP Low** | **＜0.001** | **0.429** |  |  |
| **WHO IV BSP High** | **＜0.001** | **0.006** | **0.006** |  |

**Table S3b Comparison of overall survival time among different groups of patients when stratified by BSP expression and tumor grade**

| **Group**  **(Median OS)** | **WHO III BSP Low**  **(46 months)** | **WHO III BSP High**  **(20 months)** | **WHO IV BSP Low**  **(12 months)** | **WHO IV BSP High**  **(11 months)** |
| --- | --- | --- | --- | --- |
| **WHO III BSP Low** |  |  |  |  |
| **WHO III BSP High** | **0.017** |  |  |  |
| **WHO IV BSP Low** | **＜0.001** | **0.342** |  |  |
| **WHO IV BSP High** | **＜0.001** | **0.007** | **0.010** |  |
